# Chemical profile changes during pile fermentation of Qingzhuan tea affect inhibition of α-amylase and lipase

**DOI:** 10.1038/s41598-020-60265-2

**Published:** 2020-02-26

**Authors:** Lin Feng, Panpan Liu, Pengcheng Zheng, Liang Zhang, Jie Zhou, Ziming Gong, Yongchao Yu, Shiwei Gao, Lin Zheng, Xueping Wang, Xiaochun Wan

**Affiliations:** 1 Institute of Fruit and Tea, Hubei Academy of Agricultural Sciences, 430064 Wuhan, China; 2 State Key Laboratory of Tea Plant Biology and Utilization, School of Tea and Food Science & Technology, Anhui Agricultural University, 230036 Hefei, China; 3 College of Horticulture, Northwest A&F University, 712100 Yangling, Shanxi China

**Keywords:** Biochemistry, Biological techniques

## Abstract

Qingzhuan tea (QZT), a post-fermented tea, has been reported to have anti-obesity and anti-hyperglycemic effects, perhaps due to bioactive compounds that inhibit lipase and α-amylase. It is unknown what chemical constituents’ changes and what bioactive compounds occur during the manufacture of QZT. The aim of this study was to determine the secondary metabolites changes that occur during post-fermentation and how these changes affect the ability of QZT to inhibit the activities of lipase and α-amylase. During the processing steps, metabolites levels and their inhibitory effects on lipase and α-amylase were assessed. Changes in content and activities suggested that the first turn over or the second turn over was critical for the formation and conversion of bioactive compounds responsible for the anti-obesity and hypoglycemic effects. The relationship between constituents and activities was further evaluated by correlation analysis, which showed that amino acids and flavonoids might be responsible for the anti-obesity and anti-hyperglycemic effects of QZT. This study clarified that compounds were altered during pile fermentation of QZT and tentatively identified the bioactive compounds formed during QZT manufacture.

## Introduction

Qingzhuan tea (QZT) is an important Chinese dark tea, mainly produced in Hubei province. It has long been an indispensable beverage for people in high-fat and high-calorie diet areas, such as the Mongolia, Xinjiang, Qinghai and Gansu provinces of China^1^. QZT is made through five processing steps. Briefly, one bud and five leaves are plucked, fixed at high temperature, rolled, dried in the sun, then fermented in a large pile, which is turned three times and aged for several months. After this pile fermentation, the tea is steamed, pressed and dried to obtain the Qingzhuan dark tea (QZT)^2,3^. Like other dark teas, pile fermentation is critical for the formation of the characteristic flavor and quality of QZT^2,4^. During this process, oxidation, condensation, degradation, and polymerization of polyphenols and amino acids occur through the joint actions of damp heat and microorganisms to form the pure aroma and mellow taste of QZT^3,5,6^. The current research on QZT fermentation has mainly focused on fluorine removal, leaf structural changes, polysaccharide content, and microbial sequencing^3,7–9^. However, little research has been done on the chemical changes during pile fermentation of QZT, which has led to the process not being accurately controlled and thus varied quality of QZT. Tea manufacturing is an indispensable condition for the formation of quality components and functional ingredients. Different tea types have unique quality characteristics and health effects due to different processing techniques^10^. By adjusting and optimizing the tea processing techniques, metabolic flux could be altered, quality ingredients and functional ingredients transformations can be purposefully activated, thereby improving tea quality components formation and health effects^11^. Therefore, the analysis of the variation in chemical constituents during pile fermentation of QZT processing may help to elucidate the formation mechanisms of tea quality and health functions, and also provide a theoretical basis for QZT quality control.

Obesity is a major public health problem, and highly related to metabolic diseases, such as hyperlipidemia, hyperglycemia and cardiovascular disease^12^. Excessive intake of carbohydrates and lipids is the leading cause of obesity. Some digestive enzyme inhibitors, such as acarbose or orlistat, have been used to treat diabetes or obesity by limiting the absorption of carbohydrates or lipids^13,14^. However, these chemically synthesized inhibitors have some gastrointestinal side effects. Therefore, there is an ongoing search for natural digestive enzyme inhibitors with fewer side effects for prevention or mitigation the symptom of obesity or diabetes. Previous researches have shown that some types of tea exhibited pronounced inhibitory effects towards the activities of α-amylase and pancreatic lipase^15–18^. There are few reports on whether QZT, produced under a different process, inhibits pancreatic α-amylase and lipase activities *in vitro*, and it is unknown what components within QZT contribute to anti-obesity and anti-hyperglycemic bioactivities. In studies on other types of tea, both targeted and untargeted metabolomics approaches have been followed^19–21^. Applying liquid chromatography tandem mass spectrometry (LC-MS) and multivariate statistical analysis together, the complexity and variability of the metabolites present in tea can be revealed^22–24^. In this study, we used high-performance liquid chromatography (HPLC) and a non-targeted metabolomics approach to determine the dynamic metabolic changes that occur during QZT processing and to identify the bioactive compounds found during processing and in the final tea product. Tea extracts from different processes was tested for inhibition of pancreatic α-amylase to determine whether the anti-obesity effect of QZT is mainly caused by slowing down the digestion of starch through inhibiting α-amylase activity. In addition, it was suggested that QZT might prevent fat absorption by inhibiting pancreatic lipase similar to Pu’er tea^25^. To explore this hypothesis, water extract of each pile fermentation step samples were investigated by an *in vitro* assay of pancreatic lipase inhibition. To sum up, the purpose of this study was to clarify the effect of pile fermentation on the chemical content and the anti-obesity and anti-hyperglycemic health effects of QZT.

## Materials and Methods

### Tea processing

**Figure 1:**
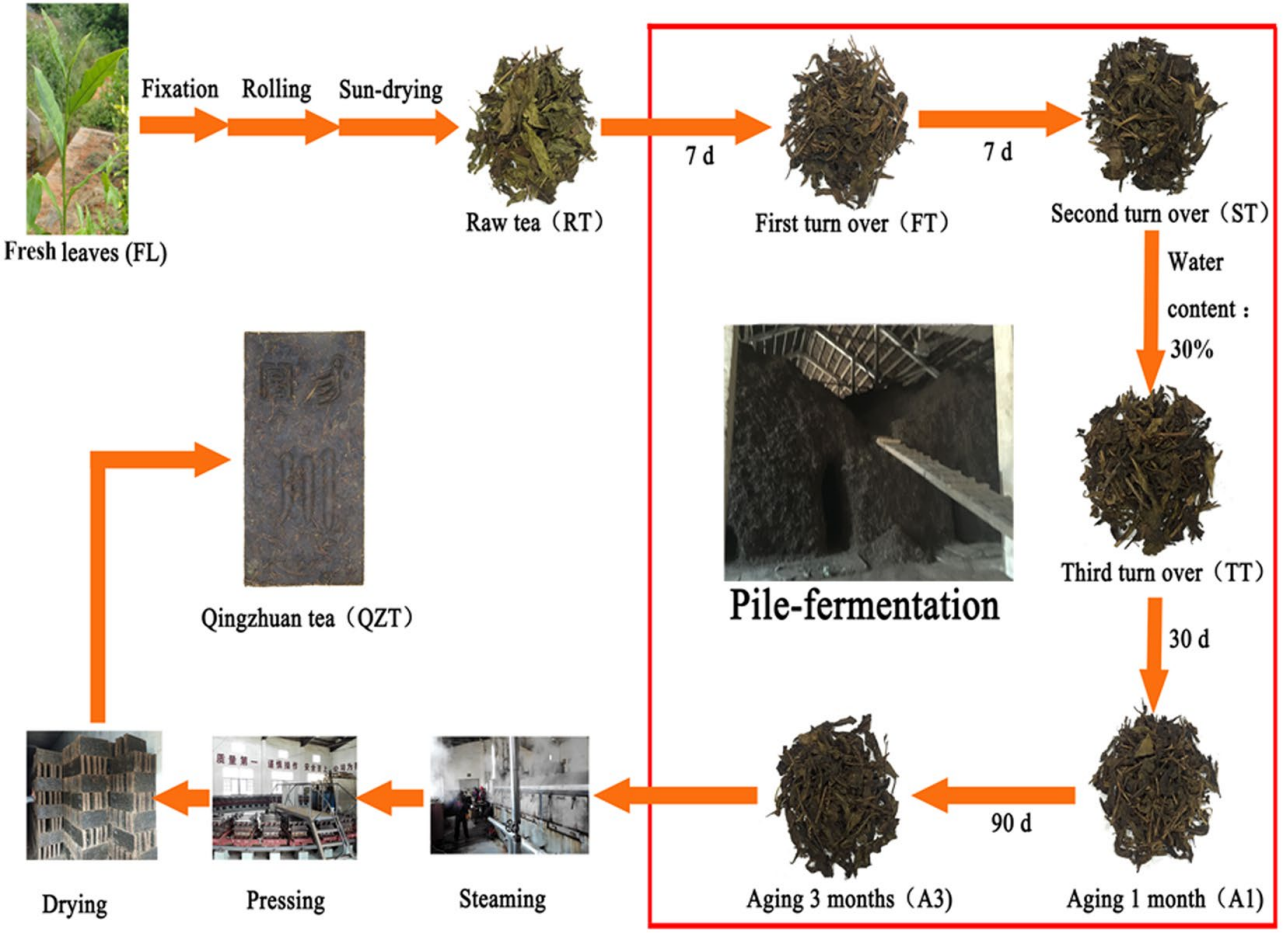
The main manufacturing steps used to produce Qingzhuan tea.

Fresh tea leaves (FL) comprising one bud with five leaves were harvested from the cultivars grown around Zhaoliqiao tea factory at Chibi City (Fig. 1). The leaves were fixed at 300 °C for 2 min, rolled for 10 min, then dried in the sun to less than 13% water content to obtain the raw tea (RT). The RT was wetted to 30% humidity and piled into 3 m tall, 2 m wide piles on the floor of an warehouse for continuous fermentation with three turnings and samples taken at 7 days (first turn over, FT), 14 days (second turn over, ST), 21 days (third turn over, TT), 51 days (aged for 1 month, A1), and 111 days (aged for 3 month, A3). The A3 samples were also steamed, pressed and dried to obtain the Qingzhuan tea (QZT). In order to ensure relative consistency, representativeness, and accuracy of the experiments, five experimental samples (5 kg) were collected from the same batch of QZT, immediately freeze-dried (FreeZone^®^ plus Freeze dryer, LABCONCO, America), and stored at −20 °C until analysis.

### Samples and chemicals

Standards of 18 amino acids and L-theanine (purity >99%), caffeine (CAF, >98%), (−)-gallocatechin (GC, >98%), (−)-epicatechin (EC, >98%), (+)-catechin (C, >98%), (−)-epigallocatechin (EGC, >98%), (−)- gallocatechin gallate (GCG, >98%), (−)-epicatechin gallate (ECG, >98%), (−)-epigallocatechin gallate (EGCG, >98%), gallic acid (GA, >98%), Pancreatic α-amylase and pancreatic lipase were bought from Yuanye Biotechnology Company (Shanghai, China). DL-4-chlorophenylalanine was bought from Sigma Chemical Co. (St. Louis, MO). Methanol, acetonitrile and formic acid were bought from Thermo Fisher (Thermo Scientific, USA).

### Sample preparation

For LC-MS analysis, the freeze-dried tea powder (50 mg) was weighed into a 1.5 mL centrifuge tube. After the addition of 1 mL of extract solvent (methanol-acetonitrile-water, 2:2:1), the samples were mixed with ultrasonication at 60 Hz and 25 °C for 20 min. The supernatants were gained after centrifuging at 12000 rpm at 4 °C, and transferred to a sample vial for LC-MS analysis.

For pancreatic α-amylase and lipase inhibition assays, the freeze-dried tea samples were ground into powder. Tea powder (0.1 g) was extracted with 5 mL of distilled water at 100 °C for 45 min while stirring, yielding a tea infusion. The supernatants were sieved through a 0.22 μm aqueous filter (Jinteng, Tianjin, China) before analysis.

### Determination of polyphenols and caffeine

Tea polyphenols and purine alkaloids were extracted using the method described by Ma *et al*., with minor modifications^26^. In brief, 3 mL of 70% methanol was added to tea powder (0.1 g) in a 5 mL centrifuge tube and vortexed at room temperature for 10 min. After centrifugation at 6000 rpm for 5 min, the sediment was extracted twice using the same method. The supernatants were collected together and diluted in 5 mL 70% methanol. The combined samples were filtered using a 0.22 μm organic membrane (Jinteng, Tianjin, China) before performing HPLC.

The HPLC (Waters 2695, Milford, America) was coupled with Agilent ZORBAX SB-Aq C_18_ (250×4.6 mm, 5 μm) column, UV detector (set at 280 nm) and a 2998 PDA detector. Column temperature was 40 °C. Mobile phase A was 2% (*v*/*v*) acetic acid, and mobile phase B was acetonitrile. The elution gradient was set as follows: 0 min, 93.5% A; 16 min, 15% A; 16–25 min, 25% A; 30 min, 93.5% A; and 40 min, 93.5% A. The injection volume and the flow rate were 10 μL and 1.0 mL/min. The contents of theaflavins (TFs), thearubigins (TRs) and theabrownin (TBs) were determined using spectrometric methods^27^.

### Determination of free amino acids

Amino acids concentrations were assayed following the method of Ma *et al*., but with minor changes^26^. In brief, a 0.1 g tea sample was extracted at 100 °C for 45 min in 10 mL of water; the mixture was shaken once every 15 min. The filtrates were sieved by a 0.22 μm aqueous filter (Jinteng, Tianjin, China) and analyzed by HPLC. Free amino acids were detected using a Waters 2695 HPLC system coupled with a 2998 PDA detector. Mobile phase A, B, C was Waters AccQ•Tag Eluent A, acetonitrile and water, respectively. The column temperature and wavelength were 37 °C and 248 nm. The elution gradient was set as follows: 0 min, 100% A; 0.5 min, 99% A, 1% B; 18 min, 95% A, 5% B; 19 min, 91% A, 9% B; 10.1 min, 1% B; 29.5 min, 83% A, 17% B; 33 min, 60% B, 40% C; 36 min, 100% A; and 45 min, 100% A. The injection volume and the flow rate were 10 μL and 1.0 mL/min.

Soluble sugar was assayed following the sulfuric acid-anthrone colorimetric method^28^.

### LC-MS analysis

LC-MS analysis was conducted following Wang *et al*., with some modifications^20^. LC-MS analysis was conducted using a Ultimate 3000 (Dionex, Sunnyvale, CA) coupled with an Orbitrap Elite™ hybrid ion trap-orbitrap mass spectrometer (Thermo Fisher Scientific, USA). Hyper Gold column (1.9 μm, 2.1×100 mm) was used to separate tea samples. Mobile phases A and B was 0.1% (*v*/*v*) formic acid and acetonitrile. The elution gradient was set as follows, 0 min, 5% B; 2 min, 40% B; 7 min, 80% B; 11 min, 95% B; 15 min, 95% B; 15.5 min, 5% B; 20 min, 5% B. The injection volume and the flow rate were 4 μL and 0.3 mL/min. The MS was conducted under both positive and negative modes with HESI spray voltage at 3.8 and 3.2 KV, successively. The sheath gas pressure, auxiliary gas pressure, capillary temperature was 35 arb, 10 arb and 350 °C, respectively. The full-scan MS mode with resolution 60,000 and scan range *m*/*z* 50–1000 was used for LC-MS running.

### Metabolomics analysis

The raw data collected from the LC-MS was analysed using the Qualitative Analysis Software (Thermo SIEVE 2.1) to get the peaks information of retention time, mass-to-charge ratio (*m/z*), and intensity of MS. For each chromatogram, each ion intensity was calibrated using the internal standard. The obtained data was processed by multivariate statistical analyses, including principal component analysis (PCA) and orthogonal partial least squares discriminant analysis (OPLS-DA), to determine the tea metabolites variations induced by the steps of the manufacturing process.

### Pancreatic α-amylase and lipase inhibition assays

The ability of each sample (FT through QZT) to inhibit pancreatic α-amylase was analysed using an iodine-starch kit^29^. Briefly, the 0.1 g tea power were extracted in 5 mL water, 50 μL sample and 1.0 mL starch substrate (0.4 g/L) were added to pH 7.0 PBS solution (incubation at 37 °C for 5 min), then 50 μL α-amylase (1 mg/mL) was mixed together. After incubated for 7.5 min, 1.0 mL iodine diluent (0.01 M) and 3.0 mL water was added for measuring at 660 nm. All samples were analysed with six independent repetitions. The inhibitory rate of samples on α-amylase was calculated by the following equation:

$${\rm{Inhibition}}\,{\rm{rate}}\,( \% )=({1-A}_{{\rm{sample}}}{/A}_{{\rm{blank}}})\times 100 \% $$

The ability of tea samples to inhibit pancreatic lipase was assayed by a kit following the published method^12^. Briefly, 50 μL tea sample and 1.0 mL substrate (0.4 g/L) were added to 100 mM pH 8.2 Tris-HCl buffer (incubation at 37 °C for 5 min), then 50 μL lipase (1 mg/mL) was mixed together and immediately measured the absorbance (A_1_) at 420 nm. After 20 min, the absorbance (A_2_) was measured again. All samples were analysed in six biological duplications. The inhibition rate of samples on lipase was calculated as the following equation.

$${\rm{Inhibition}}\,{\rm{rate}}\,( \% )=[1\,-\,({{\rm{A}}}_{{\rm{1sample}}}\,-\,{{\rm{A}}}_{{\rm{2sample}}})/({{\rm{A}}}_{{\rm{1blank}}}\,-\,{{\rm{A}}}_{{\rm{2blank}}})]\times 100$$

### Statistical analyses

Results were expressed as mean ± SD, with three or six separate determinations. One-Way ANOVA analysis and correlation analysis were performed by SPSS 17.0 (SPSS Inc., Chicago, IL, USA). Values in Table 1 that were labeled with different letters represent a significant difference of (*P* < 0.01). The significance level between groups in Table 2 are represented by ** for *P* < 0.01 and by * for *P* < 0.05. Heat-map analysis was analysed using Multi Experiment Viewer software (version 4.8.1).

**Table 1:** Contents of the main compounds found during Qingzhuan tea processing (mean ± SD, mg/g, DW).

Content (mg/g, DW, mean ± SD)	Pile fermentation processes							
	Fresh leaves (FL)	Raw tea (RT)	First turn over (FT)	Second turn over (ST)	Third turn over (TT)	Aging for 1 month (A1)	Aging for 3 months (A3)	Final tea product (QZT)
**Tea polyphenols**								
C	0.69 ± 0.12^d^	0.40 ± 0.01^c^	0.21 ± 0.03^b^	0.05 ± 0.01^a^	0.04 ± 0.01^a^	0.05 ± 0.01^a^	0.06 ± 0.01^a^	0.04 ± 0.01^a^
EC	7.39 ± 0.65^c^	6.31 ± 0.15^b^	1.05 ± 0.07^a^	0.63 ± 0.01^a^	0.56 ± 0.02^a^	0.74 ± 0.02^a^	0.76 ± 0.02^a^	0.59 ± 0.01^a^
EGC	22.05 ± 1.43^e^	18.91 ± 0.62^d^	3.01 ± 0.31^b^	2.26 ± 0.07^a^	2.00 ± 0.07^a^	2.64 ± 0.13^c^	2.41 ± 0.08^b^	2.72 ± 0.35^b^
CG	1.77 ± 0.18^c^	0.85 ± 0.02^a^	0.98 ± 0.04^b^	0.69 ± 0.02^a^	0.71 ± 0.04^a^	0.80 ± 0.03^a^	0.71 ± 0.04^a^	0.68 ± 0.05^a^
E	13.14 ± 0.80^c^	9.69 ± 0.20^a^	1.39 ± 0.07^a^	0.90 ± 0.02^a^	0.93 ± 0.03^a^	1.06 ± 0.05^a^	1.00 ± 0.04^a^	0.96 ± 0.04^a^
EGCG	58.07 ± 2.86^c^	39.93 ± 0.75^b^	3.52 ± 0.30^a^	2.46 ± 0.02^a^	2.69 ± 0.06^a^	2.95 ± 0.10^a^	2.93 ± 0.07^a^	2.38 ± 0.20^a^
GC	9.38 ± 1.71^c^	7.14 ± 1.43^b^	1.97 ± 0.06^a^	1.26 ± 0.15^a^	0.82 ± 0.02^a^	0.79 ± 0.01^a^	0.75 ± 0.01^a^	0.70 ± 0.01^a^
GCG	1.57 ± 0.26^d^	1.24 ± 0.01^c^	0.99 ± 0.07^b^	0.62 ± 0.03^a^	0.67 ± 0.01^a^	0.65 ± 0.03^a^	0.63 ± 0.01^a^	0.65 ± 0.02^a^
Total catechins	114.06 ± 5.17^c^	84.47 ± 3.02^b^	13.11 ± 0.80^a^	8.85 ± 0.21^a^	8.44 ± 0.17^a^	9.66 ± 0.36^a^	9.26 ± 0.17^a^	8.73 ± 0.32^a^
GA	1.35 ± 0.08^a^	2.85 ± 0.09^b^	8.79 ± 0.60^f^	6.97 ± 0.09^e^	4.64 ± 0.11^d^	3.94 ± 0.08^c^	3.60 ± 0.09^c^	3.43 ± 0.08^c^
TFs	0.14 ± 0.02^c^	0.04 ± 0.005^a^	0.07 ± 0.01^b^	0.06 ± 0.01^b^	0.03 ± 0.002^a^	0.09 ± 0.002^b^	0.07 ± 0.01^b^	0.07 ± 0.01^b^
TBs	3.18 ± 0.23^b^	5.41 ± 0.21^c^	7.42 ± 0.48^c^	2.53 ± 0.24^b^	1.45 ± 0.13^a^	1.26 ± 0.30^a^	1.33 ± 0.25^a^	1.27 ± 0.03^a^
TRs	4.64 ± 0.21^a^	4.24 ± 0.17^a^	4.12 ± 0.08^a^	5.21 ± 0.09^a^	6.52 ± 0.15^b^	7.93 ± 0.35^c^	7.50 ± 0.15^c^	7.61 ± 0.30^b^
**Purine alkaloid**								
Caffeine	28.99 ± 0.75^a^	28.79 ± 0.07^a^	27.44 ± 1.16^a^	27.49 ± 0.43^a^	27.32 ± 0.38^a^	27.40 ± 0.08^a^	27.76 ± 0.46^a^	27.27 ± 1.07^a^
**Amino acids**								
Asp	0.55 ± 0.04^d^	0.86 ± 0.01^e^	0.18 ± 0.02^c^	0.14 ± 0.04^b^	0.11 ± 0.03^a^	0.08 ± 0.02^a^	0.06 ± 0.01^a^	0.08 ± 0.01^a^
Ser	0.18 ± 0.01^b^	0.14 ± 0.04^b^	0.05 ± 0.02^a^	0.06 ± 0.03^a^	0.05 ± 0.02^a^	0.06 ± 0.01^a^	0.05 ± 0.02^a^	0.05 ± 0.02^a^
Glu	1.01 ± 0.03^b^	1.22 ± 0.03^c^	0.16 ± 0.01^a^	0.10 ± 0.02^a^	0.10 ± 0.01^a^	0.10 ± 0.02^a^	0.09 ± 0.02^a^	0.13 ± 0.01^a^
Gly	0.07 ± 0.02^b^	0.04 ± 0.01^a^	0.03 ± 0.01^a^	0.03 ± 0.01^a^	0.03 ± 0.01^a^	0.03 ± 0.01^a^	0.02 ± 0.01^a^	0.03 ± 0.01^a^
His	0.02 ± 0.01^a^	0.04 ± 0.01^a^	ND	ND	0.02 ± 0.00^a^	0.02 ± 0.01^a^	0.02 ± 0.01^a^	0.02 ± 0.00^a^
Gln	0.21 ± 0.01^b^	0.37 ± 0.12^c^	0.14 ± 0.02^a^	0.14 ± 0.02^a^	0.15 ± 0.03^a^	0.16 ± 0.03^a^	0.16 ± 0.02^a^	0.16 ± 0.02^a^
Arg	0.40 ± 0.05^b^	0.40 ± 0.03^b^	ND	ND	ND	ND	ND	ND
Thr	0.17 ± 0.01^d^	0.13 ± 0.03^c^	0.11 ± 0.02^c^	0.07 ± 0.02^b^	0.07 ± 0.02^b^	0.01 ± 0.00^a^	0.03 ± 0.01^a^	0.06 ± 0.01^b^
Ala	0.50 ± 0.04^c^	0.23 ± 0.03^b^	0.07 ± 0.01^a^	0.08 ± 0.01^a^	0.07 ± 0.01^a^	0.04 ± 0.01^a^	0.04 ± 0.01^a^	0.07 ± 0.01^a^
Pro	0.23 ± 0.09^b^	0.22 ± 0.03^b^	0.08 ± 0.02^a^	0.07 ± 0.02^a^	0.09 ± 0.01^a^	0.05 ± 0.01^a^	0.06 ± 0.02^a^	0.04 ± 0.02^a^
L-Theanine	5.66 ± 0.02^c^	5.75 ± 0.01^c^	0.17 ± 0.02^a^	0.08 ± 0.03^a^	0.15 ± 0.02^a^	0.12 ± 0.03^a^	0.12 ± 0.03^a^	0.52 ± 0.02^b^
Cys	0.11 ± 0.03^c^	0.07 ± 0.02^b^	0.04 ± 0.01^a^	0.02 ± 0.01^a^	0.02 ± 0.01^a^	0.03 ± 0.01^a^	0.02 ± 0.01^a^	0.02 ± 0.01^a^
Tyr	0.17 ± 0.03^c^	0.12 ± 0.03^b^	0.06 ± 0.02^a^	0.04 ± 0.01^a^	0.06 ± 0.01^a^	0.06 ± 0.02^a^	0.06 ± 0.01^a^	0.07 ± 0.02^a^
Val	0.08 ± 0.02^a^	0.07 ± 0.01^a^	0.37 ± 0.09^b^	0.41 ± 0.11^c^	0.39 ± 0.08^b^	0.37 ± 0.06^b^	0.36 ± 0.04^b^	0.35 ± 0.05^b^
Ornithine	0.11 ± 0.02^c^	0.16 ± 0.03^d^	0.05 ± 0.02^b^	0.02 ± 0.01^a^	0.05 ± 0.02^b^	0.05 ± 0.01^b^	0.06 ± 0.02^b^	0.05 ± 0.02^b^
Lys	0.08 ± 0.01^a^	0.06 ± 0.02^a^	0.06 ± 0.02^a^	0.06 ± 0.02^a^	0.06 ± 0.01^a^	0.06 ± 0.02^a^	0.05 ± 0.02^a^	0.05 ± 0.02^a^
Ile	0.05 ± 0.01^d^	0.04 ± 0.01^c^	0.02 ± 0.01^a^	0.03 ± 0.01^b^	0.03 ± 0.01^b^	0.02 ± 0.01^a^	0.02 ± 0.01^a^	0.02 ± 0.01^a^
Leu	0.09 ± 0.02^a^	0.06 ± 0.02^a^	0.36 ± 0.09^b^	0.46 ± 0.12^c^	0.47 ± 0.11^c^	0.39 ± 0.07^b^	0.41 ± 0.08^b^	0.38 ± 0.08^b^
Phe	0.07 ± 0.02^c^	0.05 ± 0.01^b^	0.02 ± 0.01^a^	0.02 ± 0.01^a^	0.02 ± 0.01^a^	0.02 ± 0.01^a^	0.03 ± 0.01^a^	0.02 ± 0.01^a^
Total AA	9.79 ± 0.43^b^	10.03 ± 0.60^b^	1.99 ± 0.20^a^	1.83 ± 0.15^a^	1.94 ± 0.10^a^	1.66 ± 0.09^a^	1.65 ± 0.08^a^	2.12 ± 0.02^a^
**Soluble sugar**								
Total soluble sugar	7.79 ± 0.54^b^	6.44 ± 0.37^a^	6.10 ± 0.41^a^	5.73 ± 0.29^a^	5.56 ± 0.13^a^	5.43 ± 0.54^a^	5.37 ± 0.48^a^	5.34 ± 0.29^a^

Notes: Different lowercase letters means *P* < 0.01; ND, Not detected or the content lower than 0.01 mg/g; GA, gallic acid; GC, (−)-gallocatechin; EGC, (−)-epigallocatechin; C, (+)-catechin; EC, (−)-epicatechin; EGCG, (−)-epigallocatechin gallate; GCG, (−)-gallocatechin gallate; ECG, (−)-epicatechin gallate; TFs, Theaflavins; TRs, Thearubigins; TBs, Theabrownine; Ala,Alanine; Val,Valine; Leu,Leucine; Ile, Isoleucine; Phe, Phenylalanine; Pro, Proline; Gly, Glycine; Ser, Serine; Thr, Threonine; Cys, Cysteine; Tyr, Tyrosine; Gln, Glutamine; His, Histidine; Lys, Lysine; Arg, Argnine; Asp, Aspartic acid; Glu, Glutamic acid; AA, amino acids.

**Table 2:** Correlation analyses of α-amylase and pancreatic lipase inhibition ratio and the VIP metabolites compounds identified during QZT pile fermentation processes.

Component	Correlation coefficient	
	α-amylase	Pancreatic lipase
Valine	0.85**	0.842**
Leucine	0.783**	0.85**
3-O-Methyl-L-Dopa	−0.726*	−0.658
Kaempferol 3-rhamnoside	0.889**	0.875*
Morin	0.687*	0.695*
Malvidin	0.681*	0.687*
Apigenin 7-glucuronide	−0.824**	−0.841**
Theophylline	0.766*	0.842**
Pyropheophorbide A	0.704*	0.766*
Theaspirane	−0.755*	−0.785*
Pheophorbide A	0.676*	0.598

Of the 91 VIP compounds, these 11 constituents were picked for further analysis because they showed significant correlation with α-amylase and pancreatic lipase inhibition.

Note: **p < 0.01, *p < 0.05.

## Results and Discussion

### Changes in tea catechins, catechins’ oxidation products and caffeine contents during QZT processing

Tea polyphenols and caffeine are important ingredients that affect the taste and aroma of tea. While most free amino acids have a refreshing taste, L-theanine, which accounts for about 60% of the free amino acids in tea, has an umami taste. After black tea and dark tea making critical steps, fermentation or post fermentation, the content of L-theanine is dramatically decreased or not detectable^30,31^. Therefore, it is important to detect the changes in L-theanine and other amino acids content that occur during QZT manufacturing processes (Table 1). Tea polyphenols and alkaloids are the main contributors to the taste and quality of tea infusion. The products of catechins oxidation, including theaflavins (TFs), thearubigins (TRs) and theabrownin (TBs), are the primary pigments directly or indirectly associated with the color of tea liquor and dry tea^32,33^. 8 flavan-3-ols (EGCG, EGC, ECG, GCG, EC, C, GC and CG), GA and caffeine were detected and quantified by HPLC (Table 1). In the FL, EGCG, ECG, EGC, GC and EC were the main polyphenols, while significantly decreased after the FT. Conversely, gallic acid (GA), a building block of the other catechins, increased to 8.79 ± 0.11 mg/g and remained relatively high through the following processes. TFs and TRs gradually decreased during the pile fermentation processes, and had a slightly increased in the FT process, followed by relatively stable content through the following processes. The content of TBs increased gradually during the pile fermentation processes. The caffeine content remained relative stability through the whole manufacturing processes. These results indicated that the FT had the greatest effect on catechin, TFs, TRs and GA levels.

### Free amino acids contents during QZT processing

In the FL, the main amino acids were L-theanine, alanine, glutamic acid, and aspartic acid. Among them, the content of aspartic acid, serine, glutamic acid, glutamine, arginine and L-theanine decreased sharply after the first turn over (FT) and remained stable after subsequent processes. The largest change was the decrease in L-theanine, which was 5.75 ± 0.02 mg/g in the raw tea (RT) but decreased to 0.17 ± 0.01 mg/g after the FT. Interestingly, the contents of valine and leucine increased dramatically after the FT and remained high after subsequent processes. The content of soluble sugar gradually decreased from FT process, followed by relatively stable content through the following pile fermentation processes. These results suggested that the FT is the critical step that due to the oxidation polymerization of catechins and that the increased GA and amino acids levels (valine and leucine) play an significant role in the unique chemical profile of QZT.

### Non-targeted metabolomics analysis

**Figure 2:**
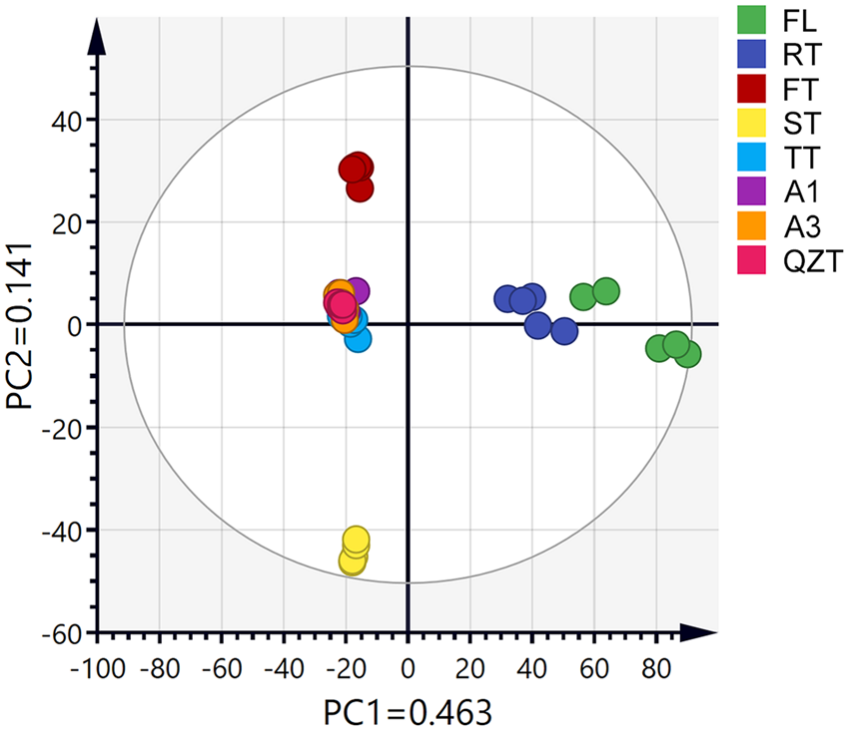
PCA score plot was used to compare the changes in the metabolic profile after each pile fermentation step during the production of Qingzhuan tea. Dried powder samples taken from fresh leaves (FL), raw tea (RT), first turn over (FT), second turn over (ST), third turn over (TT), aging for 1 month (A1), aging for 3 months (A3) and the final product of Qingzhuan tea (QZT) were analyzed. The principal components PC1 and PC2 explained 46.3% and 14.1% of the total variance, respectively.

To further reveal the dynamic changes in the chemical profile during QZT processing, non-targeted metabolomics analysis using LC-MS was applied, with the FL and QZT samples as the controls. 2709 ion features were determined after the peak alignment and the exclusion of ion features (Table S1). The raw data were analysed using a multivariate statistical software (SIMCA-P 14.1). The chemical profile after each of the eight manufacturing steps were well distinguished, all replicates from each process clustered together and separated from other samples from the other steps [Fig. 2 (PC1 = 46.3% and PC2 = 14.1%)]. The eight samples classified into five types (Fig. 2), The FL, RT, FT and ST samples all stood alone, while the other 4 samples (TT, A1, A3 and QZT) clustered together. The two initial steps, FL to RT, are similar to green tea manufacturing processes. During the FL and RT stage, with high-temperature and high-moisture, the polyphenols of fresh leaves undergo isomerization, hydrolysis and oxidation polymerization, accompanied by breakdown of starch, pectin, proteins and chlorophyll^34^. Therefore, the chemical feature of RT sample has a significant difference with the FL sample. The RT sample was the starting point of pile fermentation, during which the chemical constituents underwent major changes from RT to A3. The chemical profiles in the later fermentation stages (TT, A1 and A3) were similar to those of the final QZT, indicating that the early fermentation stages (FT and ST) were critical steps for the formation of QZT quality and taste. Therefore, the temperature, humidity and duration of the early pile fermentation steps (FT and ST) should be accurately controlled to ensure consistent quality of QZT. In addition, the contents of the TT, A1, A3 and QZT samples were similar and without significant changes, these processing steps might be shortened to decrease the production cost and improve the production efficiency of QZT. We intend to optimize these steps coupled with e-nose and taste-panel testing for further studies.

### Heat-map analysis of the relative variation in constituents during QZT processing

**Figure 3:**
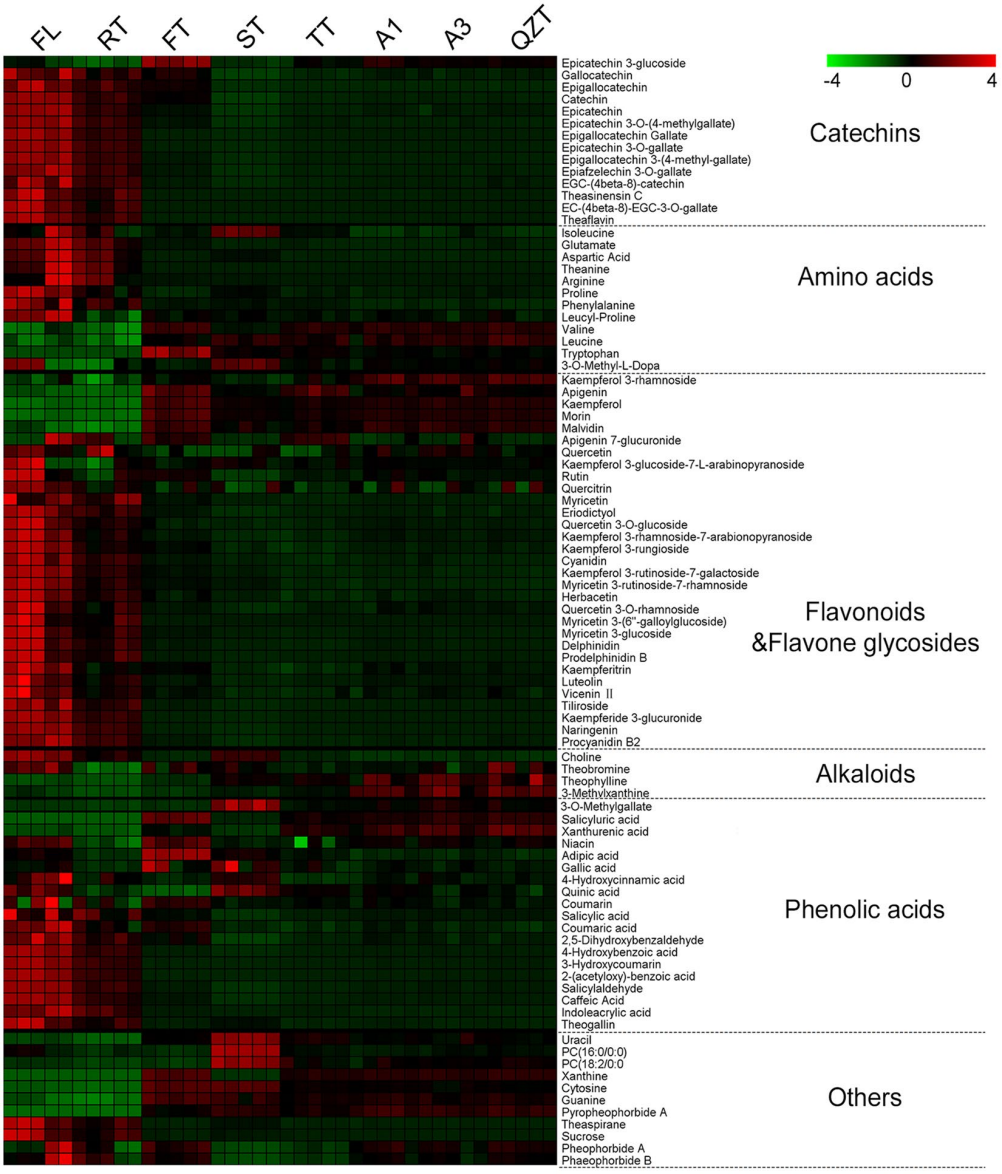
The heat-map of the annotated VIPs after different pile fermentation steps during the manufacture of Qingzhuan tea. Color-coding is graded from green to red with the relative intensity shift from low to high, respectively.

To analyse the constituents responsible for the samples clustering after each manufacturing process, OPLS-DA analysis was performed on a random combination of tea samples to find components with variable importance in the projection (VIP) scores >1 and T-test values of *P* < 0.05. A total of 91 VIPs was obtained (Fig. 3). Through comparison with standards, MS2 spectra characteristics, or matching by a combination of accurate mass and retention time, metabolomic databases, and the literatures, the VIP compounds in the samples were identified. To gain an overview of the changes in metabolites during the fermentation processes, heat-map analysis was used to reveal the relative changes of the chemical constituents. The analytical compounds were identified into 6 categories, namely catechins, amino acids, flavonoids and flavone glycosides, alkaloids, phenolic acids and other compounds, among which flavonoids were the most numerous metabolites (Fig. 3). As displayed in the heat-map, manufacturing processes either dramatically decreased or increased the content of many compounds. The following portion discuss several significant variation in each chemical category.

Tea polyphenols, such as EGCG, C, GC and their oxidation products, are the main components determining the taste of tea^35^. Tea polyphenols can be oxidized into dimeric, oligomeric and polymeric compounds during fermentation^36^. In this study, simple catechins were higher in the FL and RT samples, and were significantly reduced after the FT process, while C, EGC and GC levels gradually declined after the FT or ST process. Almost all the flavonoids and flavone glycosides decreased due to dark tea specific processing steps (after step FT), while levels of epicatechin-3-glucoside, kaempferol-3-rhamnoside, kaempferol, apigenin, quercetin, morin and malvidin increased during pile fermentation.

The content of caffeine was relatively stable, while content of theobromine and theophylline increased, mainly result from microbial fermentation. *Aspergillus niger* (van Tieghem) has been identified to produce theophylline and theobromine^4^. Meanwhile, the content of the catechins and theaflavins decreased sharply after the FT process. Some studies have pointed out that the complex of caffeine and theaflavins improves the taste and freshness of tea. Reduced caffeine and theaflavins may form complexes that are involved in forming the unique, refreshing taste of QZT.

Free amino acids are vital for tea quality, contribute to the umami taste, and also contribute to the health benefits of tea, such as L-theanine and tryptophan^37,38^. Some amino acids, such as L-theanine, aspartic acid and arginine, were significantly decreased after the FT, while leucine, valine and other branched amino acids and tryptophan and other aromatic amino acids were increased after the FT and retained a relatively high content during the later fermentation processes **(**Fig. 3**)**. Some studies have pointed out that branched amino acids can be quickly decomposed into glucose, so it was speculated that increases in essential amino acids, such as leucine, valine, and tryptophan, are sources for the taste and aroma in QZT^38,39^. Several essential amino acids exhibit special functions in the human body. For example, leucine had been proven to reduce blood glucose^39^, while tryptophan contributes to the production of melatonin, both of which are current hotspots of exploration for mechanisms contributing to obesity. Furthermore, phenolic acids, such as xanthurenic acid, salicylic acid, guanine, xanthine, cytosine, uracil, 3-methylxanthine, 16:0/0:0 and 18:2/0:0 fatty acids increased significantly after pile fermentation, and the changes in the levels of these components might have a certain correlation with the quality and beneficial effects of QZT. Moreover, the contents of phaeophorbide B, pheophorbide A and pyropheophorbide A were significantly increased after fermentation. These three compounds might contribute to the color of dry tea and tea infusion^40^.

These results suggested that, of all the manufacturing steps, the pile fermentation steps were most critical for changes in the levels of the chemical constituents. The abundant components along with the higher moisture in the early fermentation steps are conducive to a series of oxidation, condensation, degradation and polymerization reactions of tea ingredients under the joint action of humidity and microorganisms. The contents changes of these compounds before and after RT process formed the metabolic characteristics of QZT, which in turn formed the characteristics pure aroma, mellow taste and color of tea liquor and healthy active ingredients of QZT.

### Inhibitory effect of samples following pile fermentation steps on *in vitro* pancreatic α-amylase activity and pancreatic lipase (PL) activity

**Figure 4:**
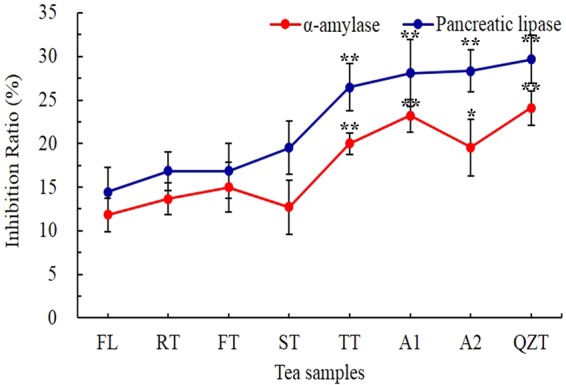
Tea infusions inhibitory effects on α-amylase and pancreatic lipase during Qingzhuan different pile fermentation processes. The results are expressed as mean ± SD (n = 6 independent replicates). Significant difference analysis with fresh leaves as the control. *P < 0.05; *P < 0.01.

A few reports have suggested that QZT possesses anti-obesity and anti-hyperglycemic effects^3,9^. It has been shown that inhibiting pancreatic lipase (PL) effectively reduces triglycerides absorption in the intestinal tract, thus preventing hyperlipidemia and obesity^41^. Inhibiting α-amylase can suppress the digestion of carbohydrates, consequently delaying the absorption of glucose and regulating the post-prandial blood glucose^42,43^. The inhibitors of α-amylase and lipase are considered to be effective in diabetes control^18^. Previous researches have shown that tea exhibited pronounced inhibitory effects on α-amylase^15,16^ and pancreatic lipase^3,17^. In our study, *in vitro* lipase and α-amylase inhibition assays were applied to reveal how the different QZT steps alter the potential hypolipidemia and hypoglycemic effects of QZT (Fig. 4).

The lipase and α-amylase inhibitory ability of tea powders prepared after each step of QZT processing were tested. Results showed that all samples following each of the steps had inhibitory effects on lipase and α-amylase (Fig. 4). Samples after the TT, A1, A3 and QZT steps exerted greater inhibitory effects than those after FL, RT, FT and ST, suggesting that the FT or ST step was an important period for formation of the bioactive compounds responsible for the health benefits of QZT, including the anti-obesity and anti-hyperglycemic effects. In addition, the content or composition of the active ingredients responsible for anti-obesity and anti-hyperglycemic effects in the TT, A1, A3 and QZT samples were significantly different from those in the FL, RT, FT and ST samples. The conversion of active ingredients or changes in content after pile fermentation may lead to different effects on anti-obesity and anti-hyperglycemic effects and are key to the formation of quality and health effects of QZT.

Furthermore, we analyzed the correlation between the inhibition of lipase and of α-amylase activities and VIP compounds (Table 2**)**. Valine, leucine, kaempferol-3 -rhamnoside, morin, malvidin, theophylline, pyropheophorbide A and pheophorbide A showed significant positive correlations with lipase and α-amylase inhibition, while 3-*O*-methyl-L-DOPA, apigenin-7-glucuronide, and the aspirane showed significant negative correlations. The results were consistent with other research studies, which have shown that flavonoids^25,44,45^ and leucine^46,47^ have positive effects on inhibiting metabolic syndrome-associated enzymes such as α-amylase, pancreatic lipase and α-glucosidase. More work is required to validate the relationship between these compounds and α-amylase and pancreatic lipase.

## Conclusion

Overall, our findings suggested that pile fermentation, more specifically the early fermentation stages (FT and ST), are the critical steps for formation of the constituents determining QZT quality and pharmacological functions. These changes in chemical composition were correlated to changes in an *in vitro* assay of obesity-preventing bioactivities (inhibition of lipase and α-amylase). Based on this study, we propose that amino acid and flavonoids might be responsible for the anti-obesity and anti-hyperglycemic effects of QZT, possibly resulting from the prevention of dietary fat absorption by inhibiting pancreatic α-amylase and lipase. Based on these results and previous studies, it seems to be worthwhile to optimize pile fermentation conditions which maximize production of these compounds while preserving desirable quality and favor profiled. Future studies are also needed to elucidate the unannotated chemical phenotypes of QZT and to determine if QZT containing higher levels of these compounds are acceptable to consumers or have greater health benefits.

## Supplementary information

Table S1.

Table S2.
