# Supplementary material for: Chemical profile changes during pile fermentation of Qingzhuan tea affect inhibition of α-amylase and lipase
Source: Sci Rep. 2020 Feb 26;10:3489. doi: 10.1038/s41598-020-60265-2 (PMC7044205; doi:10.1038/s41598-020-60265-2)
Supplement: Supplementary file 2 — Table S2. [file 41598_2020_60265_MOESM2_ESM.docx]

**Chemical profile changes during pile fermentation of Qingzhuan tea affect inhibition of α-amylase and lipase**

Lin Feng^a,b^, Panpan Liu^a^, Pengcheng Zheng^a^, Liang Zhang^b^, Jie Zhou^c^, Ziming Gong^a*^,Yongchao Yu^b^, Shiwei Gao^a^, Lin Zheng^a^,Xueping Wang^a^, Xiaochun Wan^b*^

^a^ Institute of Fruit and Tea, Hubei Academy of Agricultural Sciences, 430064 Wuhan, China

^b^ State Key Laboratory of Tea Plant Biology and Utilization, School of Tea and Food
Science & Technology, Anhui Agricultural University, 230036 Hefei, China

^c^ College of Horticulture, Northwest A&F University, 712100 Yangling, Shanxi, China
Corresponding author: Dr. Ziming Gong (ziminggong@163.com) and Dr. Xiaochun Wan (xcwan@ahau.edu.cn), Tel/Fax: +86 551 6578 6765

**Table S1** Relative abundance of chemicals following each step of QZT pile fermentation.

**Table S2** Tentative features annotation through tandem mass spectrometry and /or authentic standards

**Table S2** Tentative features annotation through tandem mass spectrometry and /or authentic standards

| ID | Name | Accurate Molecular Weight | Theoretical Molecular Weight | Delta ppm | RT （min） | Formula | Fragments | | | | | |
| --- | --- | --- | --- | --- | --- | --- | --- | --- | --- | --- | --- | --- |
| 1 | Choline* | 103.0994 | 103.0997 | 2.9 | 0.87 | C5H13NO | 60 | 58 |  |  |  |  |
| 2 | Cytosine* | 111.0429 | 111.0433 | 3.6 | 0.90 | C4H5N3O | 95 | 67 |  |  |  |  |
| 3 | Uracil* | 112.0269 | 112.0273 | 3.6 | 0.93 | C4H4N2O2 | 70 | 68 |  |  |  |  |
| 4 | Proline* | 115.0630 | 115.0633 | 2.6 | 0.88 | C5H9NO2 | 70 | 68 |  |  |  |  |
| 5 | Valine* | 117.0786 | 117.079 | 3.4 | 0.90 | C5H11NO2 | 72 | 55 | 57 |  |  |  |
| 6 | Salicylaldehyde* | 122.0364 | 122.0368 | 3.3 | 3.72 | C7H6O2 | 95 | 77 |  |  |  |  |
| 7 | Niacin* | 123.0317 | 123.032 | 2.4 | 0.92 | C6H5NO2 | 77 | 95 |  |  |  |  |
| 8 | Leucine* | 131.0942 | 131.0946 | 3.1 | 0.91 | C6H13NO2 | 86 | 69 |  |  |  |  |
| 9 | Isoleucine* | 131.0943 | 131.0946 | 2.3 | 1.53 | C6H13NO2 | 86 | 69 |  |  |  |  |
| 10 | Aspartic Acid* | 133.0370 | 133.0375 | 3.8 | 0.89 | C4H7NO4 | 72 | 104 |  |  |  |  |
| 11 | 4-Hydroxybenzoic acid* | 138.0312 | 138.0317 | 3.6 | 3.72 | C7H6O3 | 95 | 121 | 109 |  |  |  |
| 12 | Salicylic acid* | 138.0312 | 138.0317 | 3.6 | 3.44 | C7H6O3 | 121 | 95 |  |  |  |  |
| 13 | 2,5-Dihydroxybenzaldehyde* | 138.0312 | 138.0317 | 3.6 | 2.05 | C7H6O3 | 111 | 93 |  |  |  |  |
| 14 | Coumarin* | 146.0362 | 146.0368 | 4.1 | 3.75 | C9H6O2 | 91 | 103 | 77 |  |  |  |
| 15 | Adipic acid | 146.0574 | 146.0579 | 3.4 | 0.90 | C6H10O4 | 147 | 103 |  |  |  |  |
| 16 | Glutamate* | 147.0527 | 147.0532 | 3.4 | 0.89 | C5H9NO4 | 84 | 102 | 56 |  |  |  |
| 17 | Guanine* | 151.0489 | 151.0494 | 3.3 | 0.92 | C5H5N5O | 135 | 110 |  |  |  |  |
| 18 | Xanthine* | 152.0329 | 152.0334 | 3.3 | 0.93 | C5H4N4O2 | 110 | 82 |  |  |  |  |
| 19 | 3-Hydroxycoumarin | 162.0310 | 162.0317 | 4.3 | 3.72 | C9H6O3 |  |  |  |  |  |  |
| 20 | 4-Hydroxycinnamic acid* | 164.0468 | 164.0473 | 3.0 | 3.95 | C9H8O3 | 119 | 105 | 147 |  |  |  |
| 21 | Coumaric acid* | 164.0469 | 164.0473 | 2.4 | 3.57 | C9H8O3 | 91 | 119 | 147 |  |  |  |
| 22 | Phenylalanine* | 165.0785 | 165.079 | 3.0 | 2.13 | C9H11NO2 | 120 | 103 | 93 |  |  |  |
| 23 | 3-Methylxanthine* | 166.0486 | 166.0491 | 3.0 | 1.49 | C6H6N4O2 | 124 | 96 | 94 | 79 |  |  |
| 24 | Gallic acid*# | 170.0209 | 170.0215 | 3.5 | 1.37 | C7H6O5 | 153 | 127 | 125 | 109 |  |  |
| 25 | Theanine*# | 174.0999 | 174.1004 | 2.9 | 1.20 | C7H14N2O3 | 158 | 129 | 84 |  |  |  |
| 26 | Arginine* | 174.1112 | 174.1117 | 2.9 | 0.83 | C6H13N3O3 | 158 | 60 | 71 | 140 |  |  |
| 27 | 2-(acetyloxy)-benzoic acid | 180.0416 | 180.0423 | 3.9 | 3.71 | C9H8O4 |  |  |  |  |  |  |
| 28 | Caffeic acid* | 180.0416 | 180.0423 | 3.9 | 3.45 | C9H8O4 | 135 | 117 | 145 | 163 |  |  |
| 29 | Theophylline*# | 180.0641 | 180.0647 | 3.3 | 3.27 | C7H8N4O2 | 124 | 96 |  |  |  |  |
| 30 | Theobromine*# | 180.0641 | 180.0647 | 3.3 | 2.06 | C7H8N4O2 | 138 | 153 | 110 |  |  |  |
| 31 | 3-O-Methylgallate* | 184.0366 | 184.0372 | 3.3 | 3.19 | C8 H8 O5 |  |  |  |  |  |  |
| 32 | Indoleacrylic acid* | 187.0628 | 187.0633 | 2.7 | 3.49 | C11H9NO2 | 170 | 142 | 115 |  |  |  |
| 33 | Quinic acid* | 192.0627 | 192.0634 | 3.6 | 0.91 | C7H12O6 | 85 | 93 | 127 |  |  |  |
| 34 | Theaspirane | 194.1665 | 194.1671 | 3.1 | 3.96 | C13H22O |  |  |  |  |  |  |
| 35 | Salicyluric acid | 195.0526 | 195.0532 | 3.1 | 3.53 | C9H9NO4 | 152 | 95 |  |  |  |  |
| 36 | Tryptophan* | 204.0903 | 204.0893 | 4.9 | 0.88 | C11H12N2O2 | 118 | 146 | 188 | 159 |  |  |
| 37 | Xanthurenic acid | 205.0377 | 205.0375 | 1.0 | 0.90 | C10H7NO4 | 160 | 132 |  |  |  |  |
| 38 | 3-O-Methyl-L-Dopa | 211.0837 | 211.0845 | 3.8 | 0.93 | C10H13NO4 | 149 | 153 | 166 | 125 | 93 |  |
| 39 | Leucyl-Proline | 228.1466 | 228.1474 | 3.5 | 0.93 | C11H20N2O3 |  |  |  |  |  |  |
| 40 | Apigenin* | 270.0519 | 270.0528 | 3.3 | 4.66 | C15H10O5 | 145 | 119 | 91 |  |  |  |
| 41 | Naringenin* | 272.0674 | 272.0685 | 4.0 | 3.89 | C15H12O5 | 153 | 147 | 119 |  |  |  |
| 42 | Kaempferol* | 286.0466 | 286.0477 | 3.8 | 4.72 | C15H10O6 | 258 | 153 | 121 | 165 | 213 |  |
| 43 | Luteolin* | 286.0466 | 286.0477 | 3.8 | 3.93 | C15H10O6 | 153 | 135 | 241 | 161 |  |  |
| 44 | Cyanidin* | 286.0466 | 286.0477 | 3.8 | 3.80 | C15H10O6 | 137 | 213 | 109 | 241 |  |  |
| 45 | Eriodictyol* | 288.0622 | 288.0634 | 4.2 | 3.41 | C15H12O6 | 271 | 289 | 137 | 153 | 261 | 121 |
| 46 | Epicatechin*# | 290.0778 | 290.079 | 4.1 | 3.72 | C15H14O6 | 139 | 123 | 147 | 207 | 179 |  |
| 47 | Catechin*# | 290.0779 | 290.079 | 3.8 | 3.57 | C15H14O6 | 139 | 121 | 247 | 273 | 261 |  |
| 48 | Quercetin* | 302.0414 | 302.0427 | 4.3 | 3.79 | C15H10O7 | 153 | 137 | 229 | 257 | 285 | 274 |
| 49 | Herbacetin* | 302.0414 | 302.0427 | 4.3 | 3.74 | C15H10O7 | 169 | 121 | 181 | 274 |  |  |
| 50 | Morin* | 302.0414 | 302.0427 | 4.3 | 4.38 | C15H10O7 | 153 | 219 | 205 | 137 | 165 |  |
| 51 | Delphinidin* | 302.0414 | 302.0427 | 4.3 | 3.85 | C15H10O7 | 229 | 257 | 201 | 125 |  |  |
| 52 | Gallocatechin*# | 306.0728 | 306.074 | 3.9 | 2.10 | C15H14O7 | 139 | 169 | 289 | 137 |  |  |
| 53 | Epigallocatechin*# | 306.0728 | 306.074 | 3.9 | 3.40 | C15H14O7 | 127 | 169 | 141 | 139 | 181 |  |
| 54 | Myricetin* | 318.0363 | 318.0376 | 4.1 | 3.75 | C15H10O8 | 153 | 165 | 273 | 290 |  |  |
| 55 | Malvidin* | 330.0727 | 330.074 | 3.9 | 4.69 | C17H14O7 | 242 | 287 | 213 |  |  |  |
| 56 | Sucrose* | 342.1149 | 342.1162 | 3.8 | 0.88 | C12H22O11 | 203 | 185 |  |  |  |  |
| 57 | Theogallin* | 344.0731 | 344.0743 | 3.5 | 1.38 | C14H16O10 | 193 | 299 | 153 | 237 |  |  |
| 58 | Epiafzelechin 3-O-gallate* | 426.0937 | 426.0951 | 3.3 | 3.62 | C22H18O9 | 139 | 153 | 107 | 121 | 409 |  |
| 59 | Kaempferol 3-rhamnoside* | 432.1042 | 432.1056 | 3.2 | 3.84 | C21H20O10 | 287 | 269 | 257 | 147 |  |  |
| 60 | Epicatechin 3-O-gallate*# | 442.0885 | 442.09 | 3.4 | 3.89 | C22H18O10 | 139 | 153 | 123 | 425 | 291 | 273 |
| 61 | Apigenin 7-glucuronide | 446.0835 | 446.0849 | 3.1 | 3.99 | C21H18O11 | 269 | 100 |  |  |  |  |
| 62 | Quercitrin | 448.0989 | 448.1006 | 3.8 | 3.73 | C21H20O11 |  |  |  |  |  |  |
| 63 | Quercetin 3-O-rhamnoside | 448.0990 | 448.1006 | 3.6 | 3.93 | C21H20O11 | 301 | 300 | 271 |  |  |  |
| 64 | Epicatechin 3-glucoside | 452.1305 | 452.1319 | 3.1 | 3.55 | C21H24O11 |  |  |  |  |  |  |
| 65 | Epicatechin 3-O-(4-methylgallate) | 456.1041 | 456.1056 | 3.3 | 4.01 | C23H20O10 |  |  |  |  |  |  |
| 66 | Epigallocatechin Gallate*# | 458.0831 | 458.0849 | 3.9 | 3.72 | C22H18O11 | 289 | 127 | 307 | 139 | 151 |  |
| 67 | Quercetin 3-O-glucoside* | 464.0940 | 464.0955 | 3.2 | 3.74 | C21H20O12 | 300 | 137 | 229 | 153 | 301 |  |
| 68 | Epigallocatechin 3-(4-methyl-gallate) | 472.0990 | 472.1006 | 3.4 | 3.84 | C23H20O11 |  |  |  |  |  |  |
| 69 | Kaempferide 3-glucuronide | 476.0937 | 476.0955 | 3.8 | 3.72 | C22H20O12 |  |  |  |  |  |  |
| 70 | Myricetin 3-glucoside* | 480.0887 | 480.0904 | 3.5 | 3.75 | C21H20O13 | 316 | 317 | 287 | 271 | 178 |  |
| 71 | PC(16:0/0:0)* | 495.3309 | 495.3325 | 3.2 | 8.09 | C24H50NO7P | 184 | 104 |  |  |  |  |
| 72 | PC(18:2/0:0)* | 519.3308 | 519.3325 | 3.3 | 7.62 | C26H50NO7P | 263 | 221 | 337 | 417 | 88 |  |
| 73 | Pyropheophorbide A | 534.2613 | 534.2631 | 3.4 | 10.58 | C33H34N4O3 |  |  |  |  |  |  |
| 74 | Theaflavin* | 564.1251 | 564.1268 | 3.0 | 4.10 | C29H24O12 | 427 | 139 | 259 | 163 | 271 |  |
| 75 | Kaempferol 3-rhamnoside-7-arabionopyranoside | 564.1459 | 564.1479 | 3.5 | 3.70 | C26H28O14 |  |  |  |  |  |  |
| 76 | Procyanidin B2* | 578.1406 | 578.1424 | 3.1 | 3.62 | C30H26O12 | 127 | 409 | 291 | 427 | 301 | 287 |
| 77 | Kaempferitrin* | 578.1615 | 578.1636 | 3.6 | 3.78 | C27H30O14 | 287 | 285 | 415 | 433 | 147 | 329 |
| 78 | Kaempferol 3-glucoside-7-alpha-L-arabinopyranoside | 580.1404 | 580.1428 | 4.1 | 3.64 | C26H28O15 |  |  |  |  |  |  |
| 79 | Pheophorbide A | 592.2664 | 592.2686 | 3.7 | 10.20 | C35H36N4O5 |  |  |  |  |  |  |
| 80 | Epigallocatechin-(4beta->8)-catechin | 594.1352 | 594.1373 | 3.5 | 3.47 | C30H26O13 |  |  |  |  |  |  |
| 81 | Tiliroside* | 594.1353 | 594.1373 | 3.4 | 3.38 | C30H26O13 | 287 | 577 | 147 | 105 | 269 | 431 |
| 82 | Vicenin Ⅱ* | 594.1563 | 594.1585 | 3.7 | 3.64 | C27H30O15 | 577 | 559 | 445 | 427 |  |  |
| 83 | Kaempferol 3-rungioside | 594.1563 | 594.1585 | 3.7 | 3.74 | C27H30O15 | 285 |  |  |  |  |  |
| 84 | Phaeophorbide B* | 606.2457 | 606.2478 | 3.5 | 8.90 | C35H34N4O6 | 589 | 561 | 571 | 547 |  |  |
| 85 | Prodelphinidin B | 610.1301 | 610.1323 | 3.6 | 0.94 | C30H26O14 |  |  |  |  |  |  |
| 86 | Theasinensin C* | 610.1302 | 610.1323 | 3.4 | 2.28 | C30H26O14 | 139 | 611 | 593 | 307 | 247 |  |
| 87 | Rutin* | 610.1512 | 610.1534 | 3.6 | 3.79 | C27H30O16 | 303 | 465 | 129 |  |  |  |
| 88 | Myricetin 3-(6''-galloylglucoside) | 632.0979 | 632.1013 | 5.4 | 3.47 | C28H24O17 |  |  |  |  |  |  |
| 89 | Epicatechin-(4beta->8)-epigallocatechin 3-O-gallate | 746.1451 | 746.1483 | 4.3 | 3.65 | C37H30O17 |  |  |  |  |  |  |
| 90 | Kaempferol 3-rutinoside-7-galactoside | 756.2082 | 756.2113 | 4.1 | 3.80 | C33H40O20 |  |  |  |  |  |  |
| 91 | Myricetin 3-rutinoside-7-rhamnoside | 772.2029 | 772.2062 | 4.3 | 3.74 | C33H40O21 |  |  |  |  |  |  |

The compounds were identified either by MS^2^ spectra^*^ or by authentic standards^#^.
